# Supplementary material for: Presymptomatic plasma biomarkers in autosomal dominant Alzheimer’s disease: sequence and timing
Source: J Neurol Neurosurg Psychiatry. 2026 Aug 24;97(11):e339037. doi: 10.1136/jnnp-2026-339037 (PMC13691725; doi:10.1136/jnnp-2026-339037)
Supplement: Supplementary file 1 [file jnnp-97-11-s001.pdf]

Supplementary Figures and Tables to: Presymptomatic plasma biomarkers in autosomal dominant Alzheimer's disease: sequence and timing

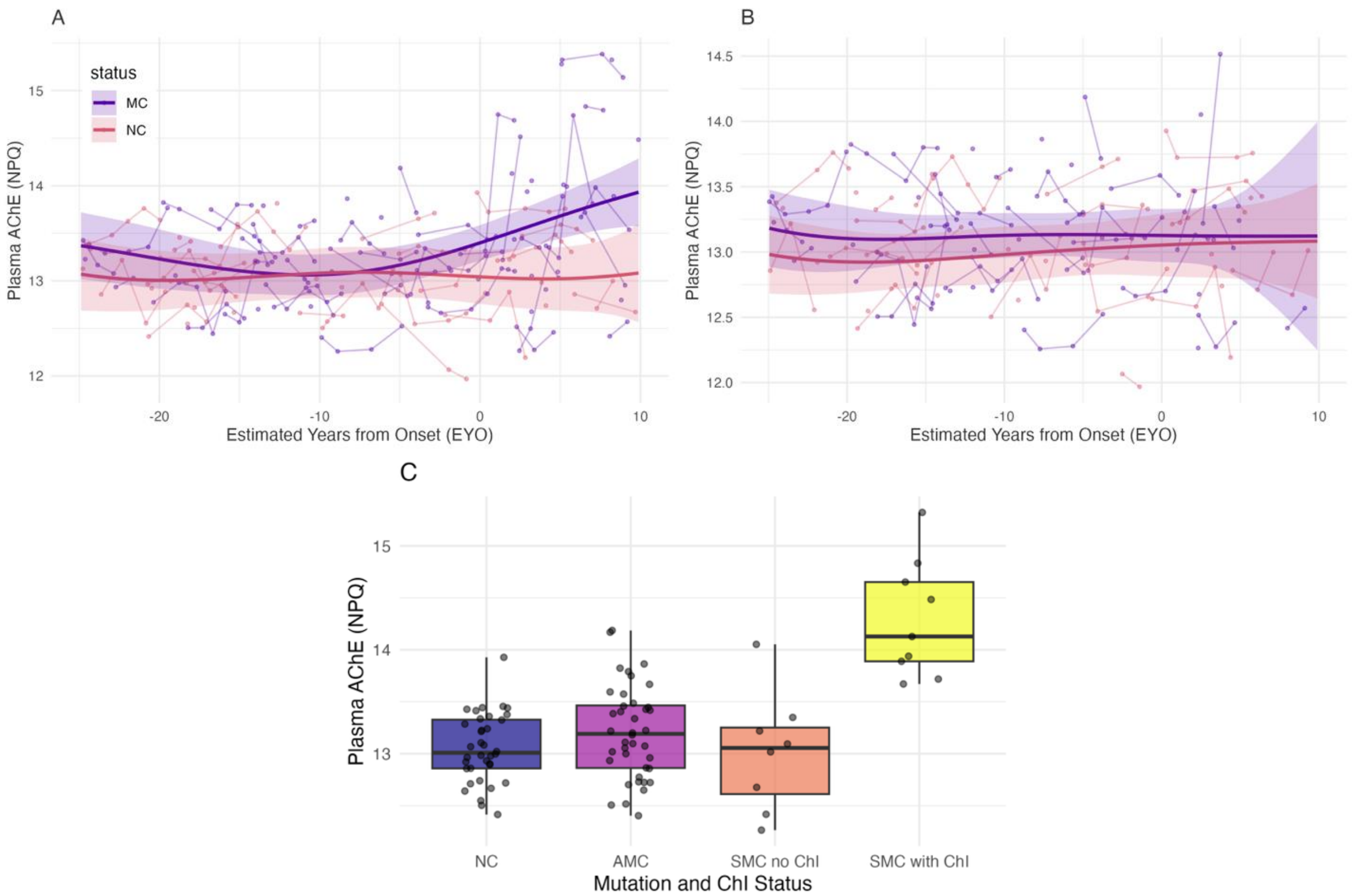

Supplementary Figure 1 – Plasma acetylcholinesterase (AChE) levels by estimated years from onset and clinical status. A and B - Coloured lines represent model-based predictions from linear mixed models, and shaded ribbons represent 95% CIs, stratified by mutation status. Observed data-points are superimposed with lines connecting repeated measures per individual. X axes are truncated and jitter has been applied to prevent inadvertent identification of individuals. A – full longitudinal cohort. B – cohort not taking cholinesterase inhibitors (ChI). C – box plots comparing cross-sectional plasma AChE levels stratified by mutation and ChI status with datapoints superimposed with jitter. MC: mutation carrier. NC: non-carrier. AMC: asymptomatic mutation carrier. SMC: symptomatic mutation carrier.

Supplementary Table 1 – Proteins analysed by the NULISA CNS 120 Panel in this analysis

Symbol	Full Protein Name
AChE	Acetylcholinesterase
AGRN	Agrin
ANXA5	Annexin A5
APOE	Apolipoprotein E
APOE4	Apolipoprotein E isoform 4
ARSA	Arylsulfatase A
Aβ38	Amyloid-beta precursor protein (Aβ38)
Aβ40	Amyloid-beta precursor protein (Aβ40)
Aβ42	Amyloid-beta precursor protein (Aβ42)
BACE1	Beta-secretase 1
BASP1	Brain acid-soluble protein 1
BDNF	Brain-derived neurotrophic factor
CALB2	Calretinin (Calbindin 2)
CCL11	Eotaxin (C-C motif chemokine ligand 11)
CCL13	C-C motif chemokine ligand 13
CCL17	C-C motif chemokine ligand 17
CCL2	C-C motif chemokine ligand 2
CCL22	C-C motif chemokine ligand 22
CCL26	C-C motif chemokine ligand 26
CCL3	C-C motif chemokine ligand 3
CCL4	C-C motif chemokine ligand 4
CD40LG	CD40 ligand
CD63	Tetraspanin-30 / CD63
CHI3L1	Chitinase-3-like protein 1
CHIT1	Chitotriosidase-1
CNTN2	Contactin-2
CRH	Corticotropin-releasing hormone
CRP	C-reactive protein
CSF2	Granulocyte-macrophage colony-stimulating factor (GM-CSF)
CST3	Cystatin C
CX3CL1	Fractalkine (C-X3-C motif chemokine ligand 1)
CXCL1	Growth-regulated alpha protein / C-X-C motif chemokine ligand 1
CXCL10	C-X-C motif chemokine ligand 10 (IP-10)
CXCL8	Interleukin-8 (C-X-C motif chemokine ligand 8)
ENO2	Gamma-enolase / Enolase 2
FABP3	Fatty acid-binding protein, heart / FABP3
FCN2	Ficolin-2
FGF2	Fibroblast growth factor 2
FLT1	Vascular endothelial growth factor receptor-1 (Fms-related tyrosine kinase 1)
FOLR1	Folate receptor alpha
GDF15	Growth/differentiation factor 15
GDI1	RuvB-like GTP-dissociation inhibitor 1
GDNF	Glial cell line-derived neurotrophic factor

GFAP	Glial fibrillary acidic protein
GOT1	Aspartate aminotransferase, cytoplasmic / Glutamic-oxaloacetic transaminase 1
HBA1	Hemoglobin subunit alpha
HTT	Huntingtin
ICAM1	Intercellular adhesion molecule 1
IFNG	Interferon gamma
IGF1R	Insulin-like growth factor 1 receptor
IGFBP7	Insulin-like growth factor-binding protein 7
IL10	Interleukin-10
IL12p70	Interleukin-12 (p70 heterodimer)
IL13	Interleukin-13
IL15	Interleukin-15
IL16	Pro-interleukin-16
IL17A	Interleukin-17A
IL18	Interleukin-18
IL1B	Interleukin-1 beta
IL2	Interleukin-2
IL33	Interleukin-33
IL4	Interleukin-4
IL5	Interleukin-5
IL6	Interleukin-6
IL6R	Interleukin-6 receptor subunit alpha
IL7	Interleukin-7
IL9	Interleukin-9
KDR	Vascular endothelial growth factor receptor-2 (VEGFR-2)
KLK6	Kallikrein-related peptidase 6
MAPT	Microtubule-associated protein tau
MDH1	Malate dehydrogenase 1, cytoplasmic
MME	Membrane metalloendopeptidase / Neprilysin
MSLN	Mesothelin
NEFH	Neurofilament heavy polypeptide
NEFL	Neurofilament light polypeptide
NGF	Beta-nerve growth factor / Nerve growth factor
NPTX1	Neuronal pentraxin-1
NPTX2	Neuronal pentraxin-2
NPTXR	Neuronal pentraxin receptor
NPY	Neuropeptide Y
NRGN	Neurogranin
Oligo-SNCA	Alpha-synuclein (oligomeric)
PARK7	Parkinsonism-associated deglycase / DJ-1
PDGFRB	Platelet-derived growth factor receptor beta
PDLIM5	PDZ and LIM domain protein 5
PGF	Placental growth factor
PGK1	Phosphoglycerate kinase 1
POSTN	Periostin

PRDX6	Peroxiredoxin-6
PSEN1	Presenilin-1
pSNCA-129	Phosphorylated alpha-synuclein (Ser129)
pTau-181	Microtubule-associated protein tau, phosphorylated at Thr181
pTau-217	Microtubule-associated protein tau, phosphorylated at Thr217
pTau-231	Microtubule-associated protein tau, phosphorylated at Thr231
pTDP43-409	TAR DNA-binding protein 43, phosphorylated at Ser409
PTN	Pleiotrophin
REST	RE1-silencing transcription factor
RUVBL2	RuvB-like AAA ATPase 2
S100A12	S100 calcium-binding protein A12
S100B	S100 calcium-binding protein B
SAA1	Serum amyloid A-1 protein
SFRP1	Secreted frizzled-related protein 1
SFTPD	Surfactant protein D / Pulmonary surfactant-associated protein D
SLIT2	Slit homolog 2 protein
SMOC1	SPARC-related modular calcium-binding 1
SNAP25	Synaptosomal-associated protein 25
SNCA	Alpha-synuclein (monomer)
SNCB	Beta-synuclein
SOD1	Superoxide dismutase [Cu-Zn]
SQSTM1	Sequestosome-1
TAFA5	Chemokine-like protein TAFA-5
TARDBP	TAR DNA-binding protein 43 (TDP-43)
TEK	Tie-2 / Angiopoietin-1 receptor
TIMP3	Metalloproteinase inhibitor 3 / Tissue inhibitor of metalloproteinase 3
TNF	Tumor necrosis factor
TREM1	Triggering receptor expressed on myeloid cells 1
TREM2	Triggering receptor expressed on myeloid cells 2
UCHL1	Ubiquitin C-terminal hydrolase L1
VCAM1	Vascular cell adhesion molecule 1
VEGFA	Vascular endothelial growth factor A
VEGFD	Vascular endothelial growth factor D
VGF	VGF nerve growth factor inducible (neurosecretory protein)
VSNL1	Vasodilator-stimulated phosphoprotein-like protein 1 / Visinin-like protein 1
YWHAZ	14-3-3 protein zeta/delta

Supplementary Table 2 – Autosomal dominant Alzheimer disease genetic variants represented in this cohort

**Gene**	**Variants**
Amyloid precursor protein (*APP*)	V717I, V717G, V717L, T719N, V717F, V715A, I716V
Presenilin-1 (*PSEN1*)	A79V, Y115C, Y115H, T116I, E120K, N135S, M139V, V142I, I143F, M146I, M146V, L153V, H163R, I168del (TATdel), I168dup, S170P, E184D, I202F, G206A, G206V, H214Y, A246E, L262F, P264L, P267S, R269H, R278I, F283L, E280G, P436S, Intron 4

Supplementary Table 3 – Longitudinal modelling of biomarker divergence between mutation carriers and non-carriers – sensitivity analyses

**Biomarker**	**Primary analysis**	**Sensitivity analyses**	
		Symptomatic MCs excluded	Age omitted from LMEMs
Aβ42	−26	−26	−26
pTau231	−24	−22	−22
pTau181	−23	−19	−22
pTau217	−21	−20	−19
MAPT	−19	−18	−17
GFAP	−14	−15	−14
BACE1	−14	−14	−13
NfL	−6	−9	−6

Numbers in table represent timepoints in estimated years from onset (EYO) at which mutation carriers (MCs) differed from non-carriers (NCs) based on the described linear mixed effects models (LMEM).
